# Efficacy and Safety of Sahastara Remedy Extract Capsule in Primary Knee Osteoarthritis: A Randomized Double-Blinded Active-Controlled Trial

**DOI:** 10.1155/2021/6635148

**Published:** 2021-01-18

**Authors:** Narin Kakatum, Piya Pinsornsak, Puritat Kanokkangsadal, Buncha Ooraikul, Arunporn Itharat

**Affiliations:** ^1^Student of Doctor of Philosophy (Applied Thai Traditional Medicine) Faculty of Medicine, Thammasat University, Bangkok, Pathum Thani, Thailand; ^2^Department of Orthopedics, Faculty of Medicine, Thammasat University, Bangkok, Pathum Thani, Thailand; ^3^Department of Applied Thai Traditional Medicine, Faculty of Medicine, Thammasat University, Klongluang, Bangkok, Pathum Thani, Thailand; ^4^Center of Excellence on Applied Thai Traditional Medicine Research (CEATMR), Faculty of Medicine, Thammasat University, Klongluang, Bangkok, Pathum Thani, Thailand; ^5^Department of Agricultural Food and Nutritional Science, Faculty of Agricultural Life and Environmental Sciences, University of Alberta, Alberta T6G 2P5, Edmonton, Canada

## Abstract

Sahastara (SHT) remedy is a Thai traditional medicine described in the Thai National List of Essential Medicine (NLEM) for the relief of muscle pain. The purpose of this study was to investigate the efficacy and safety of SHT remedy extract capsule for treating primary OA. A phase 2, double-blind, randomized, and controlled trial study was used to determine the clinical efficacy and safety of SHT in comparison with diclofenac for the treatment of knee OA. The outcome of reduce pain was measured from VAS, 100 meter time walk, and the WOMAC score of day 14 and day 28 which should reduce significantly when compared with day 0 and should be equal with or better than diclofenac. Blood pressure and blood chemistry values at day 14 and day 28 did not change when compared with day 0. The results found that SHT remedy ethanolic extract capsule can reduce all OA knee scores at day 14 and day 28 significantly when compared with day 0 and also no significant difference with diclofenac (*P* > 0.05). The SHT also showed safety values on blood pressure and blood chemistry. The SHT was observed that it had no serious side effect. The results of this study are the first report of using the SHT ethanolic extract capsule in the treatment of primary osteoarthritis of the knee. It can be recommended as an anti-inflammatory herbal drug for reducing pain in knee osteoarthritis patients.

## 1. Introduction

Osteoarthritis (OA) is the most common joint disease which occurs more frequently in males before the age of 45 and more frequently in females after 55 years of age. Osteoarthritis commonly occurs in the hands, feet, spine, and large weight-bearing joints, such as hips and knees. OA with no known cause is referred to as primary osteoarthritis. When the cause is known, it is referred to as secondary osteoarthritis. Cartilage changes when the dynamics of biology, biochemistry, and bone structure around it changed. In the elderly, involvement of synovial fluid is most common. The pain is an important clinical complaint [1]. The oral and topical NSAIDs are most commonly used to relieve pain in OA knee patients. Diclofenac was used as an active control which showed a superior effect for osteoarthritis [2]. Not only NSIADs were used for OA treatment but alternative medicine was also commonly used, including acupuncture, Thai massage, sesame oil, yellow oil, and herbal medicine [3–5].

Sahastara (SHT) is a remedy in the National Essential Drugs List (2011) which comprises 21 species of herbs as shown in Table 1. Previous research studies supported anti-inflammatory activity of SHT remedy. IC_50_ values of SHT ethanolic extract on nitric oxide and COX-2 inhibition were 2.81 *μ*g/ml and 16.97 *μ*g/ml, respectively [6]. SHT ethanolic extract exerted similar activity to NSAIDs. It was also found that SHT extract had no mutagenic effects on *Salmonella typhimurium* TA98 and TA100 isolated from patients [7]. The effectiveness and safety of the treatment with 3000 mg/day of powder SHT for primary osteoarthritic knee, as compared to 75 mg/day of diclofenac over 28 days, indicated that the SHT powder formulation was as effective as diclofenac. Furthermore, the SHT powder remedy was not toxic to the liver and kidney [8].

A previous study showed that SHT ethanolic extract exerted a higher inhibitory effect on nitric oxide (NO) release in activated murine macrophages cell line (RAW 264.7) than indomethacin (IC_50_ = 2.81 and 20.32 *μ*g/mL, respectively) [6]. SHT ethanolic extract and its components were also tested for anti-inflammation by their inhibitory effects on LPS-stimulated PGE_2_ release in RAW 264.7 cells [9]. The SHT extract and indomethacin (positive control) had IC_50_ values of 16.97 and 1.00 *μ*g/mL, respectively. *P. nigrum* [10, 11] and *P. retrofractum* [12, 13] which are the main ingredients (33.42%) of SHT preparation also showed high anti-inflammatory activity on PGE_2_ release with IC_50_ of 17.70 and 23.08 *μ*g/mL, respectively [6]. Piperine, a main component of both *Piper* species, exerted anti-inflammatory activity on human OA chondrocytes by inhibiting the IL-1*β* which induces the production of PGE_2_ and NO [14, 15]. Moreover, previous clinical studies on both SHT remedy (in powder form) and diclofenac had shown a significant reduction of VAS pain scores [8, 16]. The ethanolic extract of SHT in the capsule form was also studied on healthy volunteers with 300 and 600 mg/day for 28 days and showed safety on the kidney, liver, and blood [17]. The pharmacokinetic of piperine was also studied with oral administration dosing of 100–200 mg of SHT extract to healthy Thai volunteers and demonstrated that piperine was detectable in plasma for at least 48 hours with evidence of enterohepatic recirculation [18]. Thus, the objective of this research was to investigate the clinical efficacy of SHT ethanolic extract capsule compared with the conventional anti-inflammatory drug, diclofenac, in osteoarthritic knee patients and its safety over the four weeks of continuous treatment. This is the first study to report on clinical efficacy and safety of SHT ethanolic extract capsule in OA knee patients in order to determine whether further development of the SHT ethanolic extract capsule as medicine for OA is warranted. This study was also performed to determine whether the SHT ethanolic extract capsules can reduce the number of SHT powder capsules currently taken by the patients and hence increase the efficacy of the treatment.

## 2. Materials and Methods

### 2.1. Research Design

The research was a randomized, double-blind, and controlled trial (phase 2) designed to study the clinical efficacy and safety of the SHT remedy ethanolic extract capsule in comparison to diclofenac for the treatment of knee OA at Thammasat University Hospital, Pathum Thani Province, Thailand. It was approved by the Medical Ethics Committee of the Faculty of Medicine, Thammasat University, which was accepted by the Thai FDA (MTU-EC-TM-2-116_2/59). It was also registered at the Clinical Trials.gov (NCT04591795).

### 2.2. Subjects

Sixty-six outpatients from Department of Orthopedics, Thammasat University Hospital, between 40 and 70 years of age were recruited for this study. They were diagnosed with primary osteoarthritis of the knee according to the American College of Rheumatology's clinical and radiological criteria [19]. The patients who were included in this study had no plan for knee arthroplasty in three months, and their minimum pain symptom severity was ensured by the visual analogue scale (VAS) score of at least 20 mm from 100 mm. The exclusion criteria were patients rated with severe knee osteoarthritis (grade 4, based on the Kellgren and Lawrence radiographic system) [20], patients with serious medical conditions such as uncontrolled hypertension (BP > 140/90 mmHg), severe GI disease, congestive heart disease, liver and renal dysfunction, and obese patients with body mass index (BMI) more than 32 kg/m^2^.

### 2.3. Sample Size

The estimate of the sample size was based on the previous phase 2 clinical trial of the SHT powder capsule in the treatment of primary osteoarthritis [8]. The lower level of average VAS pain scores from the previous clinical trial was 44.1 (S.D. = 23.5) for SHT powder capsules and 31.8 (S.D. = 22.8) for diclofenac, based on statistical calculations with Stata computer programs at the power of 80 and type I (alpha) error of 0.05. The calculated number of volunteers for each of the two treatments was 28, thus requiring 56 people for both treatments. Allowing for a dropout of 10%, i.e., six additional volunteers, a minimum of 62 recruits would be required to satisfy the clinical trial protocol. The following equation was used in the calculation:

(1) $$\begin{array}{c} n_{1}=\frac{{\left(z_{1}-\left(\alpha /2\right)+z_{1}-\beta \right)}^{2}\left[\sigma_{1}^{2}+\left(\sigma_{2}^{2}/r\right)\right]}{\Delta^{2}},\\ r=\frac{n_{2}}{n_{1}},\\ \Delta =\mu_{1}-\mu_{2}. \end{array}$$

In this experiment, a total of 75 volunteers were screened and 66 were chosen, with 33 each allocated to SHT and diclofenac treatment groups.

### 2.4. Drug Preparation

The SHT remedy was prepared according to Thai National List of Essential Medicine 2011 (NLEM). The proportion of medicinal plant ingredients and their sources is shown in Table 1. All plants were cleaned immediately of extraneous materials, dried at 50°C, weighed according to the SHT recipe, mixed together, and ground to pass through sieve number 80. The ground SHT was macerated at room temperature with 95% ethanol (L/S = 2 : 1) for three days and filtered through a Whatman number 1 filter paper. The residue was further macerated with the same solvent two more times. The extracts were combined and concentrated with a rotary evaporator (Rotavapor R-205, Buchi, Switzerland). Quality control of all plant ingredients and SHT preparation was based on Thai herbal pharmacopoeia (appearance, chemical fingerprints, disintegration, microbial contamination, heavy metal contamination, and loss on drying). Piperine in SHT powder and extract was analyzed by high-performance chromatography (HPLC) to ensure that the piperine content in the SHT ethanolic extract was not less than 19 mg/g. The dry extract was pulverized and filled in 500 mg capsules with excipient (the concentration of SHT extract is 100 mg per capsule). Diclofenac sodium, 25 mg enteric-coated tablets (Voltaren®, Novartis), for oral administration, was filled into 500 mg capsules (1 tablet of diclofenac sodium per capsule). Lactose monohydrate as a placebo was also prepared in capsules (500 mg per capsule). Omeprazole (20 mg) (Miracid®, Berlin) was used as an open labeled medication.

### 2.5. Procedures

The patients who met the inclusion criteria were informed, signed a consent form, and divided randomly into two groups of treatment, using a computer-generated program, by an individual who did not have any contact with the investigators. The patients received a randomized number sequentially from a secret random list. Treatment assignment was also concealed from all investigators involved in the trial. Each patient received the same appearance of the treatment that contains treatment code, which was opened only in medical emergency condition. The masking was opened after data analysis. In the clinical trial, demographic data, clinical signs and symptoms, laboratory tests (complete blood count, fasting blood sugar, lipid profile, the liver function test, the renal function test, and urine analysis), visual analogue scale (VAS) for pain, 100 meter walk times, and the Western Ontario and McMaster Universities (WOMAC) index scores were collected on the first visit for baseline data and after receiving the treatment on days 14 and 28 [21, 22].

### 2.6. Drug Administration

The patients were divided randomly into two groups. Those in group 1 received the SHT extract 300 mg/day (one capsule of 100 mg SHT ethanolic extract three times daily before meals). This was the equivalent dose of SHT remedy that was indicated in NLEM at 3,000 mg/day of SHT remedy in the powder form. The patients in group 2 received diclofenac sodium 75 mg/day (1 capsule of 25 mg diclofenac three times daily after meals) [22]. In addition, the patients in both groups received 20 mg of omeprazole, 1 capsule before breakfast for the prevention of adverse gastrointestinal effects [8].

Clinical assessment: the treatments were completed in 28 days with the clinical and laboratory assessments at the 14^th^ and 28^th^ days. After treatments, a global assessment was performed on the patients at the last visit. The clinical efficacy was evaluated from the VAS pain scores, the 100 meter walk times, the WOMAC index scores (ranging from 0 to 96) at days 0, 14, and 28, and the global assessment on a 0–4 Likert scale (0, none; 4, excellent). The clinical efficacy and safety outcomes were evaluated by clinical examinations and laboratory analyses.

Toxicity of the drugs was considered in excluding patients following the USFDA guidance for the industry in the toxicity grading scale such as creatinine more than 1.7 mg/dL, BUN more than 26 mg/dL AST, and ALT more than 2.5x upper limit of normal (ULN) or ALP more than 2.0x ULN.

### 2.7. Statistical Analysis

The changes in mean values from baseline to days 14 and 28 for each group were analyzed by the repeated measured analysis of variance (ANOVA) or Friedman's test. The mean values between the two groups were compared using Student's *t* test or the Mann–Whitney *U* test. The comparison of the global assessments of the two groups was analyzed by the chi-square test, with *P* < 0.05 indicating a significant difference. SPSS software (version 16.0, USA) was used to analyze the data.

## 3. Results

**Figure 1:**
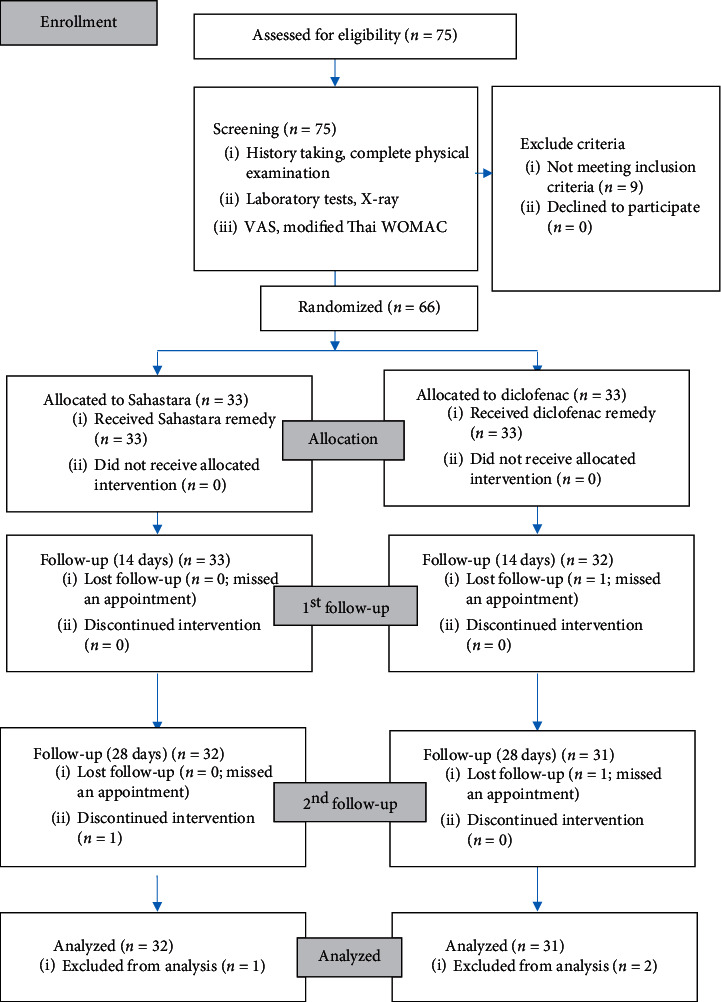
Flow chart of the study design.

In this clinical trial, 75 volunteers were screened and 66 patients were chosen and divided into two groups of 33 people each, one group receiving SHT ethanolic extract capsules and the other receiving diclofenac capsules. Both groups showed no significant differences in their baseline characteristics and the radiographic grading (Table 2). Originally, 66 patients were chosen for the study, but only 63 (95.45%) patients completed the study (32 in the SHT group and 31 in the diclofenac group). Three patients dropped out at the follow-up visits with unrelated intervention and nonserious reasons (two patients missed appointments, and one patient suffered from a traumatic wrist injury that required surgery) **(**Figure 1**).**

### 3.1. Efficacy of SHT Extract Capsule in Primary Osteoarthritis Patients

The clinical trial results showed that the SHT ethanolic extract capsule had the ability to relieve pain, reduce inflammation, improve daily life activities, decrease the WOMAC scores, and 100 meter walk times (Table 3). Comparison of all criteria (WOMAC scores on all physical index and 100 meters walk) between SHT ethanolic extract capsule and diclofenac showed no significant difference (*P* > 0.05), but the VAS score of SHT at day 28 was higher than diclofenac and was significantly different (*P*=0.01).

### 3.2. Evaluation of the Overall Treatment (Global Assessment)

To evaluate the overall effectiveness of the OA treatment with SHT extract in comparison with diclofenac (global assessment), the symptoms were monitored at day 28, using Likert scale scores, and the results showed no significant difference between the two groups (Table 4).

### 3.3. Safety Evaluation

The safety data of SHT and diclofenac groups shown as the results of blood pressure, blood chemistry of liver function, renal function, and the other blood chemistry such as complete blood count, fasting blood sugar, and lipid profile are shown in Table 5.

The systolic and diastolic blood pressure measurements were not significantly different from the baseline nor between the two groups. All patients were examined for blood urine nitrogen (BUN) and creatinine in renal function tests and for aspartate transaminase (AST), alanine aminotransferase (ALT), and alkaline phosphatase (ALP) in liver function tests at days 14 and 28. The renal function was similar in both groups when compared with their baseline values. The liver function tests of the SHT group showed no effect in the AST, ALT, and ALP values. In contrast, the AST and ALT values increased significantly after the treatment with diclofenac. The results of other blood chemistry such as complete blood count, fasting blood sugar, and lipid profile showed no significant difference between the two drug groups.

### 3.4. Adverse Effect

The adverse effects found in both groups were abdominal discomfort, 31.25% among the subjects in the SHT group and 22.58% in the diclofenac group. However, the common side effect of the SHT extract-treated group was belching, while that in the diclofenac-treated group was gastric pain (Table 6).

## 4. Discussion

The clinical efficacy of SHT might be described from the previous studies of five anti-inflammatory markers in Sahastara remedy such as piperine, ellagic acid, gallic acid, *β*-asarone, and plumbagin. Piperine as the main component of the SHT remedy was found to reduce the percentages of iNOS, elastin, and smooth muscle cells actin and was shown to decrease blood pressure from the third week of treatment [23]. The ethanolic extract of pepper showed anti-inflammatory activity in vitro, and piperine as the isolated compound was tested on interleukin 1 *β*- (IL-1*β-*) stimulated fibroblast-like synoviocytes derived from the OA knee patients [15]. Ellagic acid as an active compound could reduce inflammation on rat paw edema at 4 hours [24]. Gallic acid (10 *μ*M) isolated from *Terminalia chebula* Retz. blocked TNF-*α* and IL-6 secretion induced by PMA plus and A23187 in HMC-1 cells (68.4% inhibition in TNF-*α* and 49.8% inhibition in IL-6) [25]. *β*-Asarone from *Acorus calamus* at the dose of 50 *μ*M inhibited the production of proinflammatory cytokines, especially IL-1*β* and TNF-*α* (*P* < 0.05). [26]. Plumbagin from *Plumbago indica* inhibited inflammatory cytokine (IL-2, IL-4, IL-6, and IFN-*γ*) production in activated lymphocytes which was stimulated with con. A (5 *μ*g/ml) following which plumbagin was added at the indicated times, and the cells were cultured for 24 h at 37°C (*P* < 0.01) [27]. Asafoetida as a component in SHT has ever been reported that it showed good efficacy on reducing inflammation from dental plaque and gingivitis compared with chlorhexidine gluconate [28].

Diclofenac is an NSAID used as a control group. It is slightly better than a placebo over all of the specific treatments and increased with greater baseline pain severity (*P* < 0.001) [2].

Five parameters were measured in this study: the level of knee pain by VAS (mm) after walk on 100 meters, the 100 meters walking time, and the 3 WOMAC index scores (pain index, stiffness index, and physical function index). The levels of knee pain in the SHT extract group (*n* = 32) were reduced significantly (*p* < 0.01) in both follow-ups (14^th^ and 28^th^ days). However, the levels of knee pain in the diclofenac-treated group (*n* = 31) was significant after 28 days of treatment (*p* < 0.01). Comparison between SHT extract and diclofenac groups on the VAS score which evaluated the pain score after 100 meters walk showed that they were not significantly different at day 0 and day 14 but differed significantly at day 28 (*p* < 0.01). The results showed that SHT extract surpassed diclofenac in reducing knee pain.

The 100 meters walk durations were reduced in both follow-ups (14^th^ and 28^th^ days). However, in the SHT extract group (*n* = 32), the reduction was significant after 28 days of treatment (*p* < 0.05). Comparison between SHT and diclofenac groups showed that they were not significantly different at days 0, 14, and 28 (*p* > 0.05).

The WOMAC index scores (pain index, stiffness index, and physical function index) in both groups were reduced significantly (*p* < 0.01) in both follow-ups (14^th^ and 28^th^ days). Comparison between SHT and diclofenac groups showed that they were not significantly different at days 0, 14, and 28. These results were consistent with those in the study on knee pain using SHT powder capsules which showed significant reduction in the mean VAS pain scores sooner than diclofenac [18]. Another study on office syndrome using SHT powder capsules in comparison with diclofenac also showed equal effectiveness in reducing pain [16]. However, this study found that SHT extract showed good reduction in stiffness index, significantly different with day 0 and no significant difference with diclofenac. SHT extract showed different results when compared with SHT powder; in that, SHT extract showed better efficacy on stiffness reduction than SHT powder [5]. In addition, the total stiffness score of SHT extract decreased on days 14 and 28, equal to that of diclofenac. However, the previous result of SHT powder showed a significant difference of the total score only on day 28. These results indicated that SHT extract was more effective on OA knee patients than SHT powder. Both extract and powder of SHT showed no significant difference when compared with diclofenac [5].

For the safety evaluation, blood pressure was shown to increase in the diclofenac group by 50% [29]. This is similar to the results of this study which showed increased blood pressure and relief of pain. However, the SHT group did not show any significant change in blood pressure as indicated by the study with SHT powder drug in rat which showed no effect on blood pressure and the vasculoprotective effect in hypertensive and NO-impaired rats [30], which is similar to another SHT powder drug study in OA knee patients [7].

With regard to the adverse side effects of SHT powder and diclofenac, a previous study [8] reported that both groups developed abdominal discomfort. However, the side effect from the SHT extract group was minor (Table 6), i.e., only belching which is normally the effect of spicy taste of plant components, such as *Piper* spp., while that of diclofenac was a more serious gastric pain.

SHT ethanolic extract has no effect on blood pressure and renal and liver functions, which indicates that it is safe. Similar results have been reported previously on SHT powder. Diclofenac in this study showed an increase in the liver function value but remained within the normal range. This is similar to two previous reports [7, 18].

Important criteria for drugs to be included in the National Essential Drugs List are efficacy and human safety. SHT powder capsule is included in the National Essential Drugs List because it is shown to be a safe and effective drug for pain relief. Taking the drug in the form of powder at the dose of 6 × 500 mg capsules/day might affect patient compliance with the medication. This study used the SHT ethanolic extract capsules at a much smaller dose of 3 × 100 mg capsules/day and thus significantly reduced the number of capsules taken by the patients. This should increase the efficacy of the treatment since it would be easier for the patients to take. Moreover, taking SHT ethanolic extract which has been standardized [31] enables accurate amount of drug administration with known amount of active ingredient. All these should support the inclusion of the SHT ethanolic extract capsules in the National Essential Drugs List.

However, this research had some limitations with respect to sex of the patients, since we could not do block design to balance the numbers of female and male patients as suggested in the previous study. This was because almost all orthopedic patients in our hospital were female (more than 90%). Nevertheless, this is the first study on clinical trial phase II of SHT ethanolic extract capsule. Results from this study will form the basis for the continuation of the clinical study into phase III and will encourage further development of this drug.

## 5. Conclusion

This study is the first to evaluate the clinical efficacy and safety of SHT ethanolic extract capsules for the treatment of osteoarthritic knee patients in comparison with the conventional NSAID drug, diclofenac. The SHT ethanolic extract capsule can relieve the inflammation symptoms in OA knee patients almost as effectively as diclofenac, with less minor side effects. Therefore, it can be considered a safe alternative anti-inflammatory drug for the treatment of OA.

## Figures and Tables

**Table 1:** Medicinal plants in Sahastara remedy formulation (for 1,000 g of powder drug).

Thai name	Scientific name	Specimen voucher	Part used	Weight (g)	Source
Prik-Thai	*Piper nigrum* Linn.	SKP146161401	Fruit	240	Chanthaburi, Thailand
Jet-Ta-Mul-Plerng-Dang	*Plumbago indica* Linn.	SKP148160901	Root	224	Laos
Sa-Mhor-Thai	*Terminalia chebula* Retz.	SKP049200301	Fruit	104	Sa Kaeo, Thailand
Dee-Plee	*Piper retrofractum* Vahl.	SKP146160301	Fruit	96	Chanthaburi, Thailand
Tong-Tank	*Baliospermum montanum* Muell. A.	SKP121021301	Root	80	Kanchanaburi, Thailand

Wan-Nam	*Acorus calamus* Linn.	SKP015010301	Rhizome	88	Nonthaburi, Thailand
Has-Sa-Khun-Tade	*Kleinhovia hospita* Linn.	SKP183110801	Root	48	Kanchanaburi, Thailand
Ka-Ra-Boon	*Cinnamomum camphora* Linn.	SKP096030301	—	14	China
Dok-Chan	*Myristica fragrans* Houtt.	SKP121130601	Aril of seed	13	China
Luk-Chan	*Myristica fragrans* Houtt.	SKP121130601	Seed	12	China
Tien-Dang	*Lepidium sativum* Linn.	SKP057121901	Seed	11	India
Tien-Ta-Tuk-Ka-Tan	*Anethum graveolens* Linn.	SKP199010701	Fruit	10	India
Ma-Ha-Hing	*Ferula asafoetida* Linn.	SKP199060101	Resin	10	India
Tien-Sut-Ta-but	*Pimpinella anisum* Linn.	SKP199160101	Fruit	9	China
Tien-Khao	*Cuminum cyminum* Linn.	SKP199030301	Fruit	8	India
Jing-Jor	*Merremia vitifolia*	SKP054132201	Root	8	Kanchanaburi, Thailand
Tien-Dum	*Nigella sativa* Linn.	SKP160141901	Seed	7	China
Kote-Kag-Kra	*Anacyclus pyrethrum* (L.) DC.	SKP051011601	Root	6	China
Kote-Ka-Mao	*Atractylodes lancea* (Thunb) DC.	SKP051011201	Rhizome	5	China
Kote-Kan-Prao	*Picrorhiza kurroa* Benth.	SKP177161101	Root	4	India
Kote-Pung-Pla	*Terminalia chebula* Retz. (Gall)	SKP019200301	Gall	3	India

**Table 2:** Baseline characteristics of volunteers.

Characteristics	Sahastara (*n* = 32)	Diclofenac (*n* = 31)	*p* value^*∗*^
Female, no. (%)	29 (87.87)	30 (90.90)	0.68^C^

Age, mean (SD)	58.48 (6.98)	59.00 (6.1)	0.75^t^

Weight (Kg), mean (SD)	64.15 (7.6)	60.53 (9.9)	0.10^t^

Height (cm), mean (SD)	1.52 (0.08)	1.54 (0.06)	0.23^m^

BMI, mean (SD)	27.55 (3.45)	25.25 (4.13)	0.19^m^

Walk 100 meters			
Knee pain level VAS (mm), mean (SD)	57.84 (2.19)	54.61 (14.50)	0.28^t^
100 meters walk duration. (m/s), mean (SD)	138.86 (40.38)	130.41 (5.96)	0.44^m^

WOMAC index, mean (SD)			
Pain index	8.33 (2.23)	8.15 (4.21)	0.23^m^
Stiffness index	1.72 (1.79)	1.71 (1.95)	0.83^m^
Physical index	32.66 (8.77)	30.93 (10.83)	0.44^m^
Total score	42.72 (10.53)	40.81 (15.27)	0.27^m^

Kellgren and Lawrence X-ray grade			
Grade 1	7 (21.88)	4 (12.90)	
Grade 2	7 (21.88)	15 (48.39)	0.10^C^
Grade 3	18 (56.25)	12 (38.70)	
Total	32 (100)	31 (100)	

^*∗*^Statistical analysis. ^t^Independent sample *t* test; ^c^chi-square test; ^m^ = Mann–Whitney *U* test.

**Table 3:** Experimental results of Sahastara ethanolic extract and diclofenac.

Data	Follow-up	Treatment^*∗*^		*p* value^*∗∗*^
		Sahastara (*n* = 32)	Diclofenac (*n* = 31)	
Walk 100 meters				
Knee pain levels: VAS (mm)	Day 0	57.84 (2.19)	54.61 (14.50)	0.28^m^
	Day 14	46.12 (2.57)^††^	41.09 (11.01)	0.82^m^
	Day 28	36.46 (10.90)^††^	29.77 (8.86)^††^	0.01^m^
100 meters walk (seconds)	Day 0	138.86 (40.38)	130.41 (5.96)	0.44^m^
	Day 14	129.37 (29.28)	120.70 (4.14)^†^	0.11^m^
	Day 28	122.65 (26.46)^†^	111.51 (3.83)^†^	0.13^m^
WOMAC index scores				
Pain index	Day 0	8.25 (2.21)	7.64 (3.02)	0.23^m^
	Day 14	5.93 (2.52)^††^	5.61 (2.4)^†^	0.64^m^
	Day 28	3.81 (2.05)^††^	3.51 (1.94)^††^	0.63^m^
Stiffness index	Day 0	1.75 (1.81)	1.69 (1.81)	0.83^m^
	Day 14	0.7 (1.34)^†^	0.57 (1.11)^††^	0.44^m^
	Day 28	0.09 (0.39)^††^	0.09 (0.34)^††^	0.64^m^
Physical function index	Day 0	32.53 (8.8)	31.66 (9.8)	044^m^
	Day 14	24.87 (9.02)^††^	24.87 (9.02)^††^	0.19^m^
	Day 28	17.65 (7.78)^††^	16.92 (7.6)^††^	0.51^m^
Total score	Day 0	42.53 (1.88)	40.06 (14.22)	0.27^m^
	Day 14	32.96 (1.97)^††^	28.61 (11.41)^††^	0.21^m^
	Day 28	21.93 (1.75)^††^	19.77 (8.75)^††^	0.37^m^

^*∗*^Data represent mean (SD). ^∗∗^Statistical analysis. ^m^Mann–Whitney *U* test. For all within group statistical analysis: repeated measure ANOVA. ^†^Significant difference from day 0 within the group (*P* < 0.05); ^††^significant difference from day 0 within the group (*P* < 0.01).

**Table 4:** Overall assessment of treatments evaluated at day 28.

Evaluate overall treatment (global assessment)	Sahastara, no. (%)	Diclofenac, no. (%)	*p* value between groups
0 (none)	0 (0)	0 (0)	0.75^c^
1 (mild better)	1 (3.12)	1 (3.23)	
2 (moderate better)	11 (34.37)	8 (25.80)	
3 (very much better)	20 (62.50)	22 (70.96)	
4 (excellent)	0 (0)	0 (0)	
Total	32	31	

^*C*^Chi-square.

**Table 5:** Effect of SHT and diclofenac on blood pressure, renal functions, liver functions and blood chemistry.

Data	Follow-up	Treatment^*∗*^		*p* value^*∗∗*^
		Sahastara (*n* = 32)	Diclofenac (*n* = 31)	
Blood pressure				
Systolic (normal< 140 mmHg)	Day 0	128.39 (8.43)	129.55 (7.57)	0.57^**m**^
	Day 14	127.36 (7.63)	130.94 (8.32)^†^	0.66^**m**^
	Day 28	127.31 (7.32)	131.71 (8.09)^††^	0.01^**m**^
Diastolic (normal 90 mmHg)	Day 0	80.45 (7.25)	81.21 (6.88)	0.67^**m**^
	Day 14	80.45 (5.06)	81.81 (6.96)	0.38^**m**^
	Day 28	79.19 (5.01)	80.52 (7.40)^†^	0.02^**m**^
Renal functions				
Blood urea nitrogen, BUN (mg/dL) (normal range = 7.0–18.0)	Day 0	14.89 (4.3)	14.41 (5.87)	0.27^**m**^
	Day 14	16.68 (5.76)	14.97 (4.11)	0.40^**m**^
	Day 28	15.19 (3.90)	15.38 (5.00)	0.91^**m**^
Creatinine (normal range = 0.8–1.3)	Day 0	0.74 (0.12)	0.72 (0.22)	0.14^**m**^
	Day 14	0.76 (0.13)	0.75 (0.12)	0.41^**m**^
	Day 28	0.74 (0.15)	0.94 (1.11)	0.45^**m**^
Liver function tests				
AST (U/L) (normal range = 15–37U/L)	Day 0	24.71 (6.56)	24.03 (6.53)	0.55^**m**^
	Day 14	26.75 (10.88)	28.25 (7.7)	0.11^**m**^
	Day 28	27.59 (9.39)	29.74 (11.33)^†^	0.48^**m**^
ALT (U/L) (normal range = 30–65U/L)	Day 0	32.18 (9.55)	28.55 (8.4)	0.86^**m**^
	Day 14	35.56 (15.39)	34.06 (11.59)^†^	0.93^**m**^
	Day 28	33.87 (15.81	38.61 (20.15)^†^	0.25^**m**^
ALP (U/L) (normal range = 30–120U/L)	Day 0	74.27 (20.18)	78.96 (22.18)	0.59^**m**^
	Day 14	75.66 (18.22)	80.54 (18.24)	0.21^**m**^
	Day 28	76.09 (19.19)	82.00 (19.61)	0.19^**m**^
The result of another laboratory				
FBS (74–106 mg/dL)	Day 0	104.606 (27.06)	116.48 (61.15)	0.59^**m**^
	Day 14	105.96 (29.34)	114.18 (62.12)	0.39^**m**^
	Day 28	104.65 (29.41)	113.58 (54.26)	0.73^**m**^
HDL-cholesterol (40–60 mg/dL)	Day 0	55.66 (15.18)	58.00 (14.31)	0.48^**m**^
	Day 14	55.90 (15.51)	57.31 (15.55)	0.71^**m**^
	Day 28	57.28 (13.58)	53.70 (16.09)	0.52^**m**^
Total cholesterol (40–60 mg/dL)	Day 0	220.63 (40.34)	222.03 (44.27)	0.85^**m**^
	Day 14	214.35 (35.12)	220.65 (38.35)	0.37^**m**^
	Day 28	212.00 (37.78)	224.51 (41.71)	0.18^**m**^
LDL-cholesterol (0–150 mg/dL)	Day 0	133.39 (34.47)	132.63 (40.85)	0.82^**m**^
	Day 14	130.27 (32.02)	132.00 (32.00)	0.74^**m**^
	Day 28	130.25 (30.02)	132.54 (36.00)	0.76^**m**^
Triglyceride (0–150 mg/dL)	Day 0	152.66 (98.81)	144.64 (79.68)	0.98^**m**^
	Day 14	159.90 (114.84)	145.13 (85.90)	0.84^**m**^
	Day 28	135.09 (84.13)	158.68 (16.47)	0.53^**m**^
Live function total protein (6.4–8.2 mg/dL)	Day 0	4.60 (3.98)	3.93 (0.25)	0.44^**m**^
	Day 14	4.30 (3.80)	3.75 (0.34)	0.44^**m**^
	Day 28	4.20 (3.80)	3.76 (0.28)	0.24^**m**^
Albumin (3.4–5 mg/dL)	Day 0	3.98 (0.25)	3.93 (0.25)	0.51^**m**^
	Day 14	3.80 (0.27)	3.75 (0.34)	0.66^**m**^
	Day 28	3.80 (0.20)	3.76 (45 .28)	0.54^**m**^
Globulin (1.5–3.5 mg/dL)	Day 0	3.70 (0.30)	3.85 (0.47)	0.51^**m**^
	Day 14	3.65 (0.35)	3.76 (0.51)	0.66^**m**^
	Day 28	3.56 (0.28)	3.73 (0.48)	0.54^**m**^
Total bilirubin (0.2–1.0 mg/dL)	Day 0	0.63 (0.37)	0.53 (0.28)	0.11^**m**^
	Day 14	0.53 (0.34)	0.43 (0.16)	0.25^**m**^
	Day 28	0.52 (0.23)	0.47 (0.21)	0.13^**m**^
Direct bilirubin (0.0–0.2 mg/dL)	Day 0	0.13 (0.07)	0.14 (0.12)	0.87^**m**^
	Day 14	0.11 (0.05)	0.10 (0.03)	0.30^**m**^
	Day 28	0.10 (0.04)	0.10 (0.04)	0.75^**m**^
Total alkaline phosphatase (50–136 U/L)	Day 0	74.27 (20.18)	78.96 (22.18)	0.59^**m**^
	Day 14	75.66 (18.22)	80.54 (18.24)	0.21^**m**^
	Day 28	76.09 (19.19)	82.00 (19.61)	0.19^**m**^
CBC WBC (4.9–11.0 K/cumm)	Day 0	6.37 (1.42)	6.25 (1.67)	0.68^**m**^
	Day 14	5.83 (1.88)	5.77 (1.75)	0.92^**m**^
	Day 28	6.09 (1.43)	5.98 (1.57)	0.81^**m**^
Neutrophil (45–75%)	Day 0	52.45 (12.26)	52.45 (12.26)	0.40^**m**^
	Day 14	51.15 (9.11)	51.15 (9.11)	0.35^**m**^
	Day 28	52.00 (6.99)	52.00 (6.99)	0.39^**m**^
Lymphocyte (20–45%)	Day 0	37.93 (8.27)	37.93 (8.27)	0.54^**m**^
	Day 14	39.54 (8.341)	39.54 (8.34)	0.62^**m**^
	Day 28	37.66 (6.84)	37.66 (6.84)	0.74^**m**^
Monocyte (2–10%)	Day 0	3.52 (01.58)	3.55 (01.64)	1.00^**m**^
	Day 14	3.69 (01.9)	3.59 (01.67)	0.99^**m**^
	Day 28	3.70 (01.52)	3.48 (02.09)	0.18^**m**^
Eosinophil (4–6%)	Day 0	4.14 (3.47)	4.16 (4.04)	0.74^**m**^
	Day 14	4.26 (2.41)	4.97 (3.87)	0.70^**m**^
	Day 28	4.83 (2.48)	6.22 (4.43)	0.18^**m**^
Basophil (0-1%)	Day 0	0.53 (0.25)	0.57 (0.75)	0.33^**m**^
	Day 14	0.71 (01.17)	0.49 (0.44)	0.30^**m**^
	Day 28	0.47 (0.24)	0.42 (0.22)	0.62^**m**^
RBC (4.5–6.0 × 10^6^/cumm)	Day 0	4.40 (0.36)	4.36 (0.59)	0.25^**m**^
	Day 14	4.27 (0.65)	4.34 (0.63)	0.73^**m**^
	Day 28	4.32 (0.40)	4.48 (1.10)	0.99^**m**^
Hb (14–18 gm/dL)	Day 0	12.4 (0.98)	14.82 (17.60)	0.03^**m**^
	Day 14	12.14 (0.92)	11.66 (1.12)	0.14^**m**^
	Day 28	12.01 (1.00)	11.71 (1.13)	0.33^**m**^
Hct (41–51%)	Day 0	37.20 (2.42)	35.58 (3.08)	0.36^**m**^
	Day 14	36.92 (2.41)	35.81 (3.52)	0.35^**m**^
	Day 28	36.63 (3.08)	35.60 (3.50)	0.20^**m**^
Platelets (150–400 K/cumm)	Day 0	275.60 (58.63)	284.06 (78.23)	0.99^**m**^
	Day 14	286.90 (63.08)	283.87 (67.26)	0.61^**m**^
	Day 28	288.00 (64.26)	290.21 (68.90)	0.99^**m**^

^ ^*∗*^^Data represent mean (SD); ^∗∗^statistical analysis; ^m^Mann–Whitney *U* test. For all within group statistical analysis: repeated measure ANOVA. ^†^Significant difference from day 0 within the group (*P* < 0.05); ^††^significant difference from day 0 within the group (*P* < 0.01).

**Table 6:** Adverse reaction comparison between Sahastara and diclofenac.

Adverse events	Sahastara, no. (%)	Diclofenac, no. (%)	*p* value^C^
Gastric pain	0 (0)	7 (22.58)	0.11
Belching	10 (31.25)	0 (0)	0.001^∗∗^
Constipation	2 (6.25)	1 (3.22)	0.81
Dry lips and throat	4 (12.50)	2 (6.45)	0.65
Sweating	1 (3.12)	1 (3.22)	0.98
Dizziness	1 (3.12)	2 (6.45)	0.81

^C^Chi-square.

## Data Availability

The data supporting the findings of the study are available from the corresponding author upon request.
